# Core body temperature correlates of transition from aerobic to anaerobic metabolism in running

**DOI:** 10.7717/peerj.19686

**Published:** 2025-07-17

**Authors:** Marija Rakovac, Davor Šentija, Tošo Maršić, Vesna Babić

**Affiliations:** 1 University of Zagreb Faculty of Kinesiology, Zagreb, Croatia; 2 Faculty of Science and Education University of Mostar, Department for Kinesiology, Mostar, Bosnia and Herzegovina

**Keywords:** Gas exchange, Method comparison study, Rectal temperature, Treadmill running, Ventilatory threshold

## Abstract

**Purpose:**

We investigated core body temperature (CBT) during a graded exercise test (GXT) in comparison with gas exchange dynamics.

**Methods:**

Thirty-two active males performed a treadmill GXT (0.5 km/h increments every 30 seconds, 1.5% incline) until exhaustion. Gas exchange data and rectal temperature (T_re_) were continuously registered. Ten participants repeated the test for reliability assessment. The first and second gas exchange thresholds (VT_1_ and VT_2_) were determined by the simplified V-slope method, while CBT dynamics and eventual temperature thresholds (TT_1_ and TT_2_) were assessed according to the criteria defined in this study. Three independent evaluators determined gas exchange and temperature thresholds.

**Results:**

In 29 subjects, T_re_ increase was best fitted with a 3-phase segmented model of successively steeper slopes, with a linear relationship in all three segments (17 subjects), or in two segments, with a quadratic relationship for the remaining segment (12 subjects). The between-segment intersection points were considered as TT_1_ and TT_2_. In three participants, T_re_ was best fitted with a two-segment, single-breakpoint (TT_1_ or TT_2_) model. The evaluators’ objectivity was satisfactory for VT_1_ (α = 0.786), very high for TT_2_ (α = 0.941) and VT_2_ (α = 0.948). TT_1_ and VT_1_ were moderately correlated (*ρ* = 0.41, *p* = 0.021) while VT_2_ and TT_2_ were highly correlated (*r* = 0.78, *p* < 0.001) showing a small, yet statistically significant difference (12.95 ± 1.9 vs 13.43 ± 1.7 km/h, *p* = 0.039). However, test-retest reliability was low.

**Conclusion:**

The breakpoints in CBT increase observed during graded running may represent transitions between the three intensity domains of physical activity.

## Introduction

All physical activity can be sorted into three distinct intensity domains—moderate, heavy, and severe (Skinner & Mclellan, 1980; Antonutto & Di Prampero, 1995; Meyer et al., 2005; Binder et al., 2008). They reflect different metabolic sources and processes providing energy for muscular contraction, and are delineated by two transition points, or thresholds. The aerobic (or lactate) threshold (AeT) is the upper limit for the moderate intensity. Exceeding the AeT intensity, the body is progressively more reliant on carbohydrates *vs* fats as fuel, and blood lactate [La -] increases significantly compared to resting values. Carbon dioxide output (VCO_2_) and pulmonary minute ventilation (VE) show disproportionate increase compared to oxygen uptake (VO_2_), needed to eliminate excess CO_2_ produced by working muscles, and maintain homeostasis of CO_2_ pressure (Wasserman et al., 1973; Kindermann, Simon & Keul, 1979; Meyer et al., 2005). The upper limit for the heavy domain is the anaerobic threshold (AnT), also called respiratory compensation point (RCP) (Wasserman et al., 1973). AnT represents the highest intensity at which there is still an equilibrium between lactate production and elimination (maximal lactate steady state), and at higher intensities blood [La −] and [H +] rise inexorably and cannot be stabilized. At the AnT there is a second disproportionate increase in VCO_2_ and VE as compared to VO_2_, as well as a decrease in arterial pH and CO_2_ pressure (Rusko et al., 1986; Meyer et al., 2005). To test whether buffering of the metabolic acidosis prevents the occurrence of the AnT, Meyer et al. (2004) administered intravenous injections of bicarbonate during a ramp cycling test. The delayed (but not prevented) occurrence of AnT in their study indicates that exercise induced metabolic acidosis is, to a certain extent, causally involved in the occurrence of hyperventilation at the AnT.

This concept of three domains and two threshold intensities as delineating breakpoints has long been recognized and used both in medicine and sports (Loat & Rhodes, 1993)—in prescription of exercise intensity, monitoring of training and rehabilitation effects, performance and clinical outcome prediction, and athlete selection (Meyer et al., 2005; Faude, Kindermann & Meyer, 2009). Despite its widespread use, the concept of aerobic/anaerobic thresholds still remains a controversial topic in terms of its definition, underlying physiological mechanisms, as well as its measurement methods and even its mere existence (Brooks, 1985; Anderson & Rhodes, 1989; Loat & Rhodes, 1993; Myers & Ashley, 1997; Bosquet, Léger & Legros, 2002; Svedahl & MacIntosh, 2003; Poole et al., 2021).

In addition to the most widely used methods of threshold identification—blood lactate and gas exchange measurements (Svedahl & MacIntosh, 2003; Meyer et al., 2005)—a number of other methods have been proposed to explain and enhance the objectivity of the determination of aerobic-anaerobic transition. These include field and laboratory measurements of heart rate (Conconi et al., 1982), heart rate variability (Kaufmann et al., 2023), catecholamines in plasma (Schneider, McGuiggin & Kamimori, 1992; Chwalbinska-Moneta et al., 1996), myoelectric signals (Nagata et al., 1981; Lucía et al., 1999), ammonium ion in plasma (Buono, Clancy & Cook, 1984; Yuan et al., 2002), saliva electrolytes (Chicharro et al., 1994) or self-reported subjective measures (Bok, Rakovac & Foster, 2022).

Different research approaches reflect different hypotheses on the physiological mechanisms underlying the thresholds. The use of electromyography (EMG) to analyze the aerobic-anaerobic transition during graded exercise (Nagata et al., 1981; Moritani et al., 1984; Airaksinen et al., 1992; Mateika & Duffin, 1994; Chwalbińska-Moneta, Hanninen & Penttila, 1994; Taylor & Bronks, 1994; Lucía et al., 1997; Lucía et al., 1999; Hug et al., 2003; Frazão et al., 2021) is based on the hypothesis that the increases in EMG activity, resulting from the increased recruitment of fast twitch motor units during incremental exercise, would appear as noticeable thresholds that would correlate with the ones detected by conventional methods for determination of the aerobic-anaerobic transition.

Building on this, we formulated a hypothesis on possible temperature thresholds based on the changes in motor units recruitment during incremental exercise. Namely, it has been shown that within muscle fibers, compared to type-I isoforms, type-II specific myosin ATPase isoforms require 1.6−2.1 times more ATP per unit force production and therefore have a proportionately lower thermodynamic efficiency (Stienen et al., 1996; Reggiani, Bottinelli & Stienen, 2000; Barnes & Kilding, 2015). Moreover, an increase in intracellular free ADP, P_i_ and H^+^ occurs with increasing intensity of muscle contraction, leading to a decrease of the free energy (ΔG) for ATP hydrolysis (González-Alonso et al., 2000). Therefore, we hypothesized that a more pronounced recruitment of fast twitch motor units, and lower thermodynamic efficiency characteristic for increasing intensity of exercise and the aerobic-anaerobic transitions, would reflect in clearly detectable threshold-like changes in the increase of the core body temperature (CBT) during a ramp test in controlled environmental conditions. As the recruitment of fast-twitch motor units (especially type IIb at the intensities above the anaerobic threshold) results in an appreciable increase in the energy turnover per unit of work, a disproportionate increase in heat production might be expected, and detected as threshold-like CBT changes. Consequently, we hypothesized that with increasing exercise intensity the CBT slope changes would coincide with the changes in ventilatory parameters used to determine the first (aerobic) and the second (anaerobic) gas exchange thresholds (VT_1_ and VT_2_, respectively).

In the context of ventilatory and temperature parameters, Whipp & Wasserman (1970) were the first to explore the relationship between hyperpnea and rectal temperature during a progressive exercise test to fatigue, in normothermic and hypothermic state. Pulmonary ventilation in their study increased proportionally to VCO_2_ and was independent of the body temperature level, suggesting that during exercise body temperature cannot be considered an independent stimulus to ventilation. White & Cabanac (1995); White & Cabanac (1996) also explored the ventilatory response as a function of body temperature increase during incremental cycling. Contrary to the study of Whipp & Wasserman (1970), they (White & Cabanac, 1995; White & Cabanac, 1996) reported the occurrence of a breakpoint in the relationship between esophageal and tympanic temperatures and ventilatory equivalents for VO_2_ and VCO_2_, thus termed as core temperature threshold for increased ventilation (White & Cabanac, 1995; White & Cabanac, 1996). They assumed that hyperventilation during high-intensity exercise could be in part a thermolytic response involved in selective brain cooling.

Countless graded exercise tests are performed each day in clinical and sports diagnostics settings. Remarkably, to the best of our knowledge, despite the importance and extensive research of the CBT and thermoregulation, CBT dynamics in relation to increasing work rate have not been addressed in prior studies. Moreover, none of these studies evaluated core body temperature and its relationship with gas exchange data during a finely graded running test to exhaustion. Thus, the aim of our study was to (1) explore and characterize core body temperature response to graded treadmill exercise, and (2) compare core body temperature and gas exchange data measured concurrently during a finely graded GXT. Rectal temperature (T_re_) has been shown as a reliable and stable measure/representative of the internal body temperature (Armstrong et al., 2007; Lee et al., 2010) and it was intentionally chosen as the index of CBT in our study due to its proximity to the large muscle groups active in running. We hypothesized that with increasing exercise intensity the CBT would show a disproportionate increase (with threshold-like phenomena) coincident with the changes in ventilatory parameters used to determine the aerobic and the anaerobic thresholds.

## Materials & Methods

### Participants

Thirty-two recreationally active males (age 26.5 (6.6) yrs; height 179.2 (5.0) cm; body mass 76.8 (8.1) kg; mean (SD) for all values) participated in the study. The participants were kinesiology students and recreational runners recruited by the Diagnostic Center of the University of Zagreb Faculty of Kinesiology. The inclusion criteria were engagement in running activities at least four hours per week during the year prior to testing, experience in treadmill running, absence of any musculoskeletal or cardiovascular symptoms or diseases that might have influenced the results, normal resting body temperature on the day of the testing, and no recent exposure to high environmental temperatures. The measurements were performed during the spring and autumn months, so no seasonal acclimatization was expected. The participants were asked to refrain from strenuous physical activity at least 24 h, have a light breakfast at least 2 h before testing, and show up dressed in light clothing and running shoes. The purpose of the study and potential harms were explained, and written informed consent provided from all subjects. The study, approved by the Review Boards of the University of Zagreb Faculty of Kinesiology and School of Medicine (approved research proposal 04-3741/2-2008), conforms to the Helsinki Declaration.

### Study protocol

All subjects performed a graded exercise test on a motorized, calibrated treadmill (Run Race, Technogym, Italy) with incline set at 1.5%. Following a short warm-up and stretching procedure, the participants started walking for 3 min at three km/h. Thereafter, speed increased by 0.5 km/h every 30 s. The participants were instructed to start running at the speed of five km/h. At low running speeds the aerial (airborne) phase was not required, as just the stance leg had to be flexed in the mid-stance phase (spring-mass model). The test was performed until volitional exhaustion. The last full stage the subject could sustain was defined as the subject’s maximal speed. During recovery, the subjects walked at five km/h for 5 min. To determine the test-retest reliability, ten participants repeated the test within one week.

All tests were performed in the morning hours, in stable microclimatic conditions (air temperature 20−22 °C, relative humidity ≤ 60%, no appreciable sources of air flow) to keep the increase of the CBT proportional to the amount of metabolically produced heat (Lind, 1963).

### Acquisition and analysis of gas exchange parameters

We monitored gas exchange parameters continuously throughout the test, using an automated breath-by-breath system (Quark b^2^, Cosmed, Rome, Italy). The system includes a face mask worn by participants (Hans Rudolph, Shawnee, KS, USA), connected to a turbine with opto-electronic air flow meter. Expired air (one ml/s) passes through a capillary tube (*Nafion Permapure^®^*) to reach analyzers for O_2_ (zirconia) and CO_2_ (infrared). The gas concentrations are measured with an accuracy of ± 0.03%. Prior to each test the turbine was calibrated with a 3-L pump, and the O_2_ and CO_2_ analyzers were calibrated with a calibration gas (O_2_ 16.10%, CO_2_ 5.20%, NO_2_ rest).

Gas exchange data analysis and VT_1_ and VT_2_ assessment were performed within the Quark b^2^ software (Version 8.1a, Cosmed, Italy) as previously described (Sentija & Markovic, 2009). Briefly, determination of both thresholds is based on the accelerated rate of CO_2_ output compared to VO_2_ (simplified V-slope method) (Sue et al., 1988; Schneider, Phillips & Stoffolano, 1993). The point of the first disproportionate increase of VCO_2_ in comparison to VO_2_ indicated the oxygen uptake at VT_1_ and the corresponding speed. The point of the second disproportionate increase of VCO_2_ in comparison to VO_2_ (the intersection point of the below and above regression lines) represented the second ventilatory (anaerobic) threshold (VT_2_). When needed, threshold determination was supported with inspection of the VE, VE-VCO_2_ plot, respiratory exchange ratio (RER), and ventilatory equivalents for O_2_ (VE/VO_2_) and CO_2_ (VE/VCO_2_). The highest oxygen uptake for any 30-s period recorded in the incremental running test was defined as peak VO_2_. Three experienced evaluators detected VT_1_ and VT_2_ independently.

### Acquisition and analysis of core body temperature

Rectal temperature was measured with a single-use fast-response temperature probe connected to a datalogger (*Xplorer GLX,* PASCO Scientific, Roseville, CA, USA) with *DataStudio* software for data storage and analysis. The logger contains built-in temperature sensors with a resolution of 0.01 °C. The frequency of data collection was set at two Hz. Prior to each testing, the instrument was calibrated according to the manufacturer’s instructions. Subjects were carefully instructed to insert the temperature probe into the rectum, eight cm from the anal opening (Åstrand et al., 2003). The probe cable was fastened to the participants’ shorts and additionally secured with an elastic strap around the waist. After the test, temperature data were exported to Microsoft Excel and analyzed graphically.

**Figure 1:**
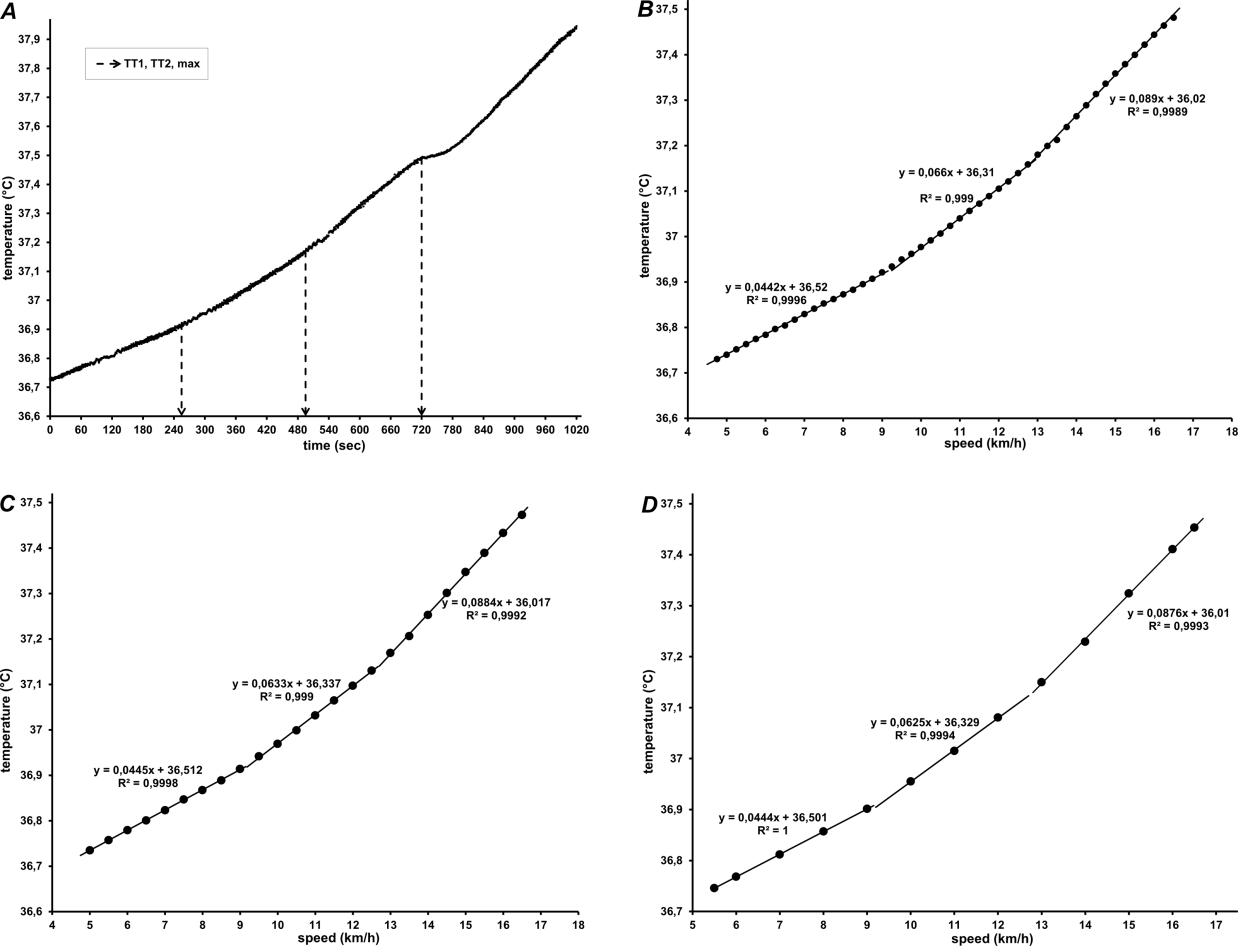
Core body temperature of one participant during graded treadmill running, in relation to (A) time (raw data), and speed averaged every 15 s (B), 30 s (C) and 60 s (D). In (A) *T*_*re*_ throughout 5 min of recovery is also shown. In (B, C, and D) regression lines with corresponding equations for the three segments are shown.

If manifested, the temperature thresholds (TT_1_ and TT_2_) were assessed by the same evaluators that detected the gas exchange thresholds. The evaluators were instructed to determine the temperature thresholds visually (by detecting breakpoints of abrupt change in the slope of the CBT-time/speed relationship) and perform computer-assisted regression analysis for confirmation. To improve the detection of the thresholds, all data were viewed and analyzed graphically as raw data, and with three different time-averaging intervals: 15-s (half-stage), 30-s (full stage), and 60-s (two-stage). Temperature data for a subject with three distinct linear phases and clear TT_1_ and TT_2_ are shown in Fig. 1.

Piecewise linear and second-order polynomial (quadratic) models were used to fit temperature data. If both the linear and polynomial functions fitted the data equally well, the lower order (linear) model was chosen. To detect trend changes and avoid overfitting, a minimum range of CBT observations for any phase was defined as four stages (2 min, or two km/h range) and a maximum number of two allowed breakpoints was selected, in line with the presumed three-domain model of physical activity. This prevented that detected breakpoints were solely affected by step-to-step changes and supported the detection of only major breakpoints in the entire intensity range. Furthermore, to identify the breakpoint as either TT_1_ or TT_2_ in cases where only one breakpoint could be discerned, it was assumed that both breakpoints are roughly equidistant from start to end of running in the test.

Evaluation of the temperature and ventilatory data was performed separately, and the evaluators were blinded for both data sets. If evaluators disagreed about a detected gas exchange or temperature threshold value, the value considered in further analyses was either: (a) the value on which two evaluators agreed (in case only one evaluator selected a different threshold value), or (b) the median value (in case all three evaluators selected different threshold values).

### Data analysis

Descriptive statistics (arithmetic mean, standard deviation and range) were calculated for each variable. The normality of data distribution was tested by the Kolmogorov–Smirnov test. The relationship between ventilatory and temperature parameters was tested using Pearson’s correlation coefficient (TT_2_ and VT_2_) and the Spearman rank correlation coefficient (TT_1_ and VT_1_). The statistical significance of the differences between ventilatory and temperature parameters was tested by the paired samples *t*-test (TT_2_ and VT_2_) and the Wilcoxon signed-rank test (TT_1_ and VT_1_). The agreement between the temperature and ventilatory thresholds was tested using regression analysis with 95% prediction and confidence intervals and by the Bland-Altman method (Altman & Bland, 1983). The objectivity of the three evaluators in identification of the ventilatory and temperature thresholds was determined by the Cronbach α coefficient. The test-retest reliability was determined by the intraclass correlation coefficient (ICC). In order to reveal the dynamics of CBT in relation to running speed and in relation to gas exchange thresholds within the whole dataset the following procedure was used: (1) the relative speed for each stage was calculated (in % of maximal speed), for each participant; (2) CBT data for all participants were added together, grouped and averaged within categories representing a relative 2% running speed increase (from 24 to 100%). Statistical significance was set at *p* ≤ 0.05. All statistical analyses were performed using Statistica, version 14.0.1.25 (StatSoft Inc., Tulsa, OK, USA).

## Results

### Modeling of core body temperature

**Figure 2:**
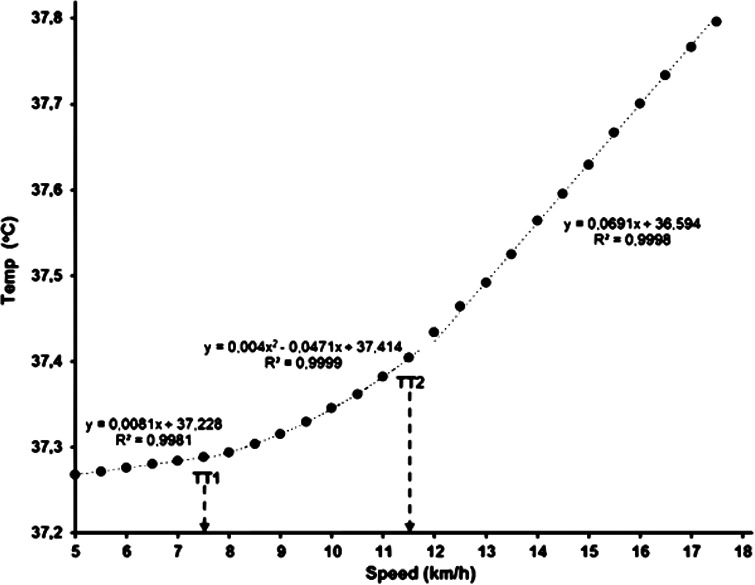
Core body temperature in relation to running speed in one participant with one quadratic (central) and two linear (initial and final) segments.

Different patterns characterized the increase of rectal temperature during graded treadmill running. The most common (detected in 17 participants) was a 3-phase segmented linear regression model, with successively steeper temperature slopes at higher speeds (Fig. 1). The second model was detected in 12 participants: the temperature data were best fitted with a quadratic relationship in one segment, and a linear relationship for the remaining two segments (Fig. 2). The breakpoints between the segments were considered as the first and second temperature thresholds (TT_1_ and TT_2_).

**Figure 3:**
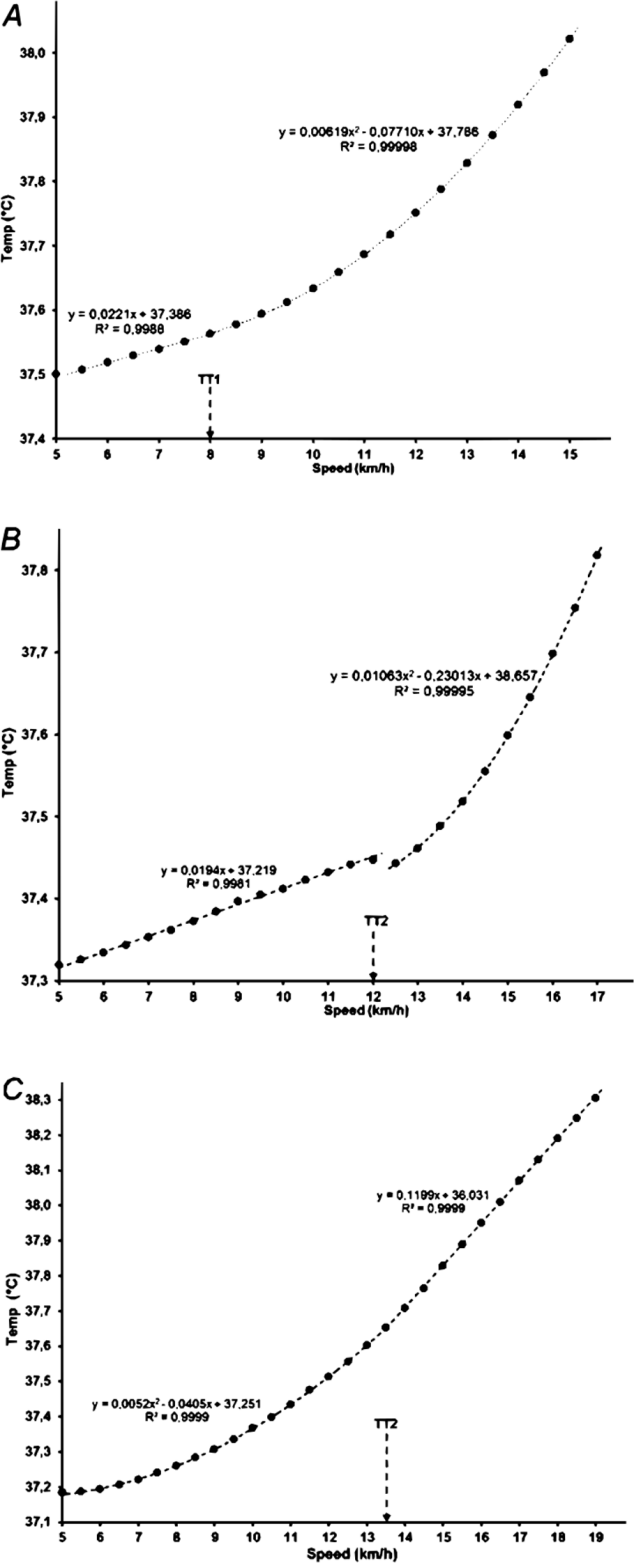
Core body temperature with a single breakpoint in relation to running speed (A, B and C showing data for three participants).

In the remaining three subjects, the temperature data were best fitted with a single breakpoint (TT_1_ or TT_2_) between two segments, one with a quadratic and one with a linear function (Fig. 3).

The mean resting rectal temperature at the start of the exercise test was 37.20 ± 0.27 °C. Initial dips in rectal temperature were observed in certain subjects, but no systemic tendency at the onset of running exercise was found. That is, T_re_ within the first (moderate) domain slowly rose in some, whilst it was preserved or even decreased in others. Rectal temperature rose with higher exertion, and continued to increase throughout the recovery period (see Fig. 1A), during which the highest values were observed in all subjects (on average, 38.42 ± 0.38 °C).

Four participants showed a sporadic drop in temperature readings lasting 1–5 s, probably caused by errors in signal transmission; the missing values of those brief periods between validly collected temperature data were easily recovered with appropriate estimated values, allowing us to construct representative core temperature profiles throughout the measurement for all participants.

### Gas exchange and temperature thresholds

**Figure 4:**
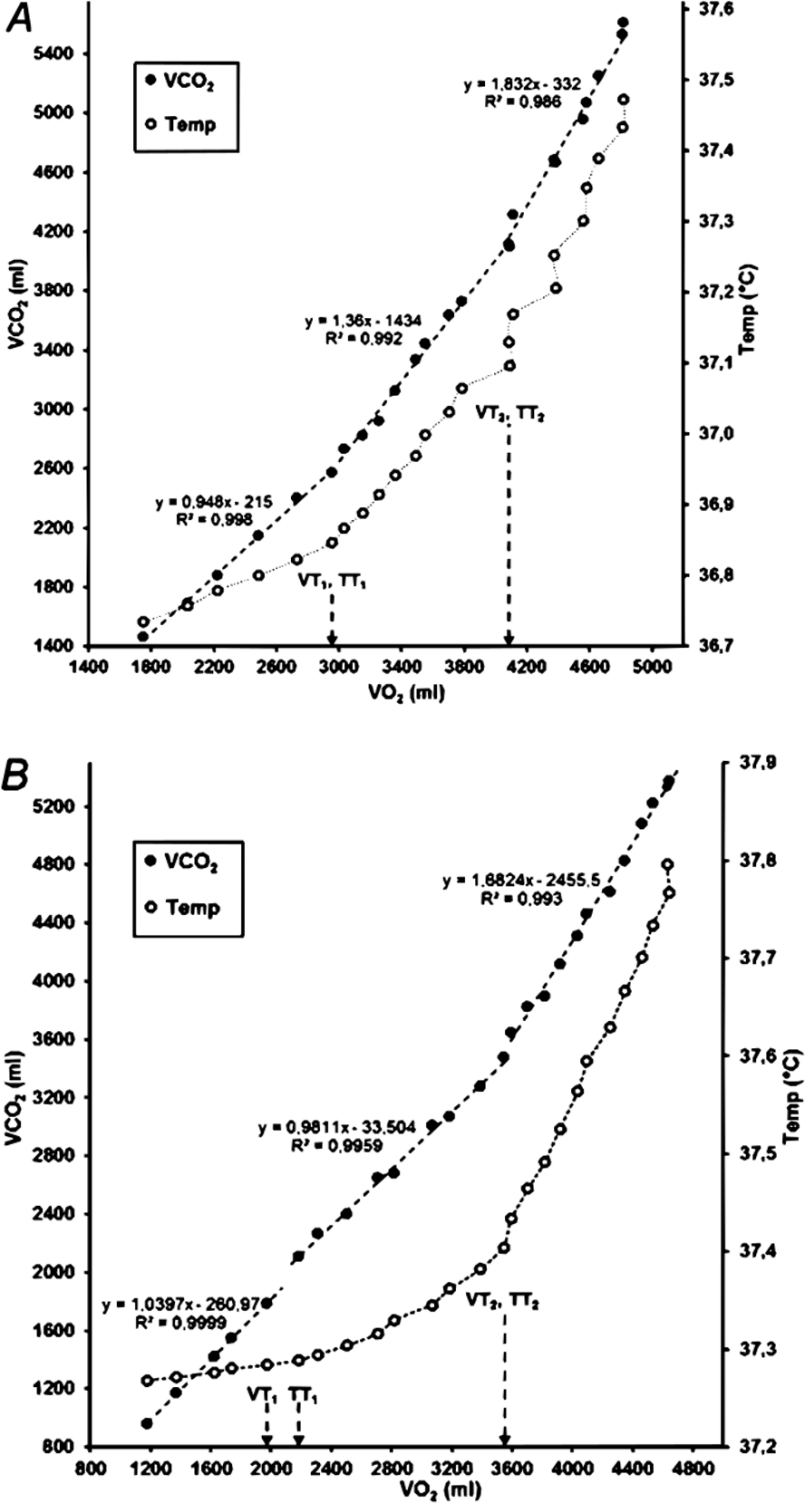
VO_2_ in relation to VCO_2_ (V-slope) and rectal temperature in two participants. Note that the marked temperature thresholds were determined from the temperature data in relation to running speed (see Fig. 1). (A) Identical gas exchange and temperature thresholds; (B) TT_1_ is one stage (0.5 km/h) higher than VT_1_.

Descriptive data of the temperature and gas exchange thresholds are presented in Table 1. Both gas exchange thresholds (VT_1_ and VT_2_) were determined from the VO_2_/VCO_2_ relationship (V-slope, Fig. 4A). However, due to artefacts in the gas exchange data, one participant was excluded from the analysis. In three participants, there was a clear breakpoint with an abrupt rise between the first and second segment in the VCO_2_/VO_2_ relationship (and was adjudicated as VT_1_), although the slope coefficient for the second segment (data between VT_1_ and VT_2_) was below 1.00 (Fig. 4B). Overall, smoother between-phase transitions and higher signal-to-noise ratio were noted for CBT, than for gas exchange parameters.

**Table 1:** Descriptive statistics of the variables at the thresholds, and at peak running speed.

	**Speed** **(km/h)**	**Temp** **(° C)**	**VO**_**2**_ **(ml/kg)**	**%VO**_**2max**_	**K-S** **p**
**TT**_**1**_	8.14 ± 0.9 (7–11)	37.33 ± 0.29 (36.61–37.83)	33.9 ± 5.0	56.8 ± 7.1	<0.01
**VT**_**1**_	8.35 ± 1.3 (6–11)	37.33 ± 0.29 (36.65–37.83)	34.5 ± 4.4	57.6 ± 5.2	>0.20
**TT**_**2**_	13.43 ± 1.7 (10.5–18)	37.59 ± 0.31 (36.92–38.13)	51.2 ± 6.1	85.4 ± 6.0	>0.20
**VT**_**2**_	12.95 ± 1.9 (9.5–17.5)	37.57 ± 0.29 (36.98–38.09)	50.3 ± 5.3	83.6 ± 3.9	>0.20
**Max**	17.78 ± 2.1 (14–22.5)	37.96 ± 0.37 (37.11–38.84)	59.9 ± 5.4	—	>0.20

**Notes.**

TT_1_ and TT_2_ first and second rectal temperature threshold VT_1_ and VT_2_ first and second gas exchange threshold Max maximal values at exhaustion Temp rectal temperature K-S p *p*-value in Kolmogorov–Smirnov test

Values are means ± standard deviation, with ranges in parentheses.

### Objectivity of the evaluators

Although noticeable in most subjects, the evaluators’ objectivity in the assessment of TT_1_ was unsatisfactory (α = 0.693). In seven participants one of the evaluators could not identify TT_1_. On the other hand, the evaluators’ objectivity in the assessment of TT_2_ was very high (α = 0.941). The objectivity of the assessment of gas exchange thresholds was satisfactory both for VT_1_ (α = 0.786) and for VT_2_ (α = 0.948).

### Relationship between temperature and gas exchange thresholds

**Figure 5:**
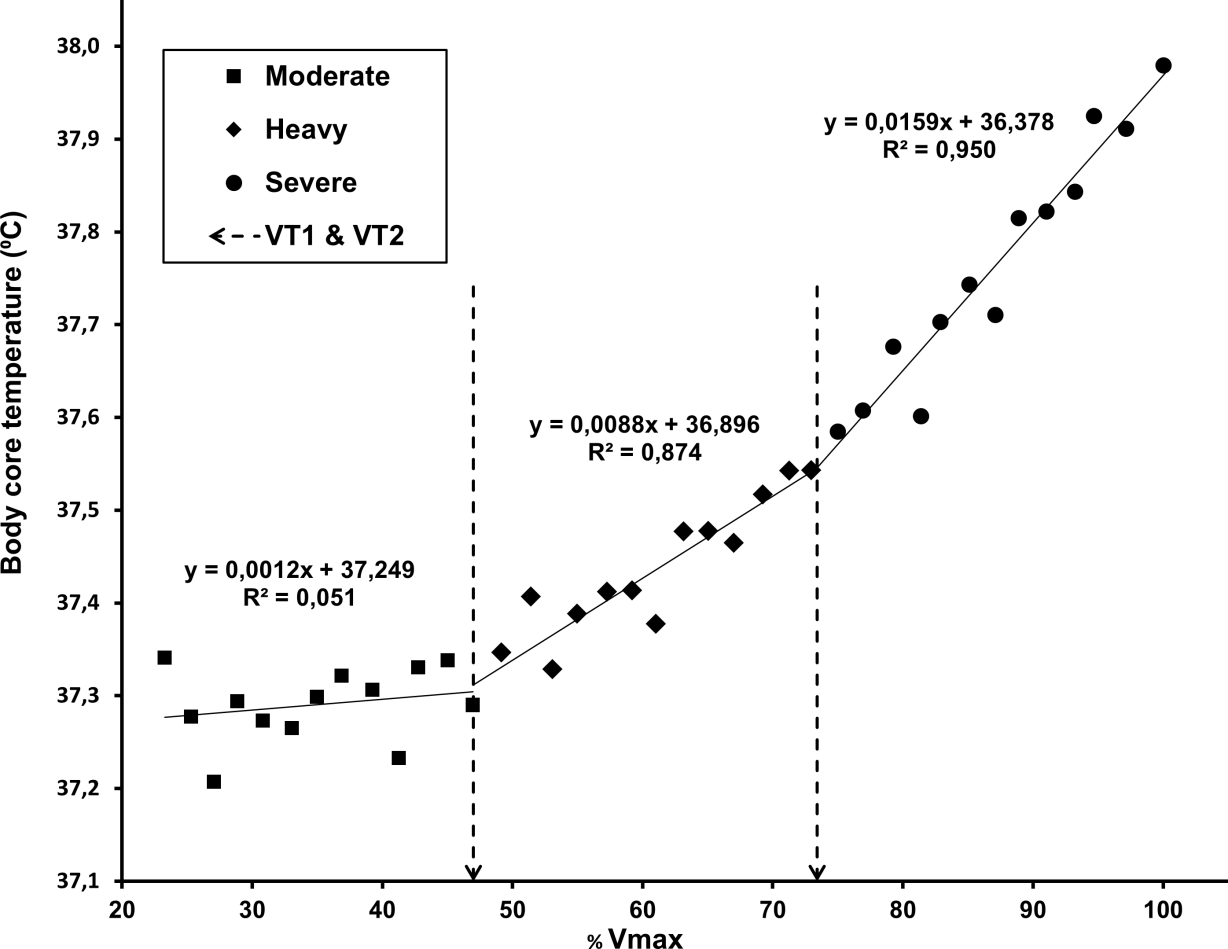
Core body temperature data for all participants, averaged in relation to the percentage of maximal running speed (2% steps). The linear regressions for the three domains were calculated with average VT_1_ and VT_2_ values as breakpoints (dashed lines).

No significant differences were found between TT_1_ and VT_1_ (*p* = 0.407), while there was a small, but significant mean difference between running speed at TT_2_ and VT_2_ (*p* = 0.039). However, when expressed as temperature, oxygen uptake or percentage of VO_2max_, the average values of VT_2_ and TT_2_ thresholds did not differ significantly (*p* > 0.10 in all cases). There was a moderate correlation between TT_1_ and VT_1_ (*ρ* = 0.41, *p* = 0.021) and a strong correlation between TT_2_ and VT_2_ (*r* = 0.78, *p* < 0.0001). In Fig. 5, rectal temperature data for all participants are presented in relation to the relative running speed, expressed as a percentage of maximal running speed (averaged at 2% steps), to visualize and confirm the presence of three CBT domains during incremental running and the similarity of breakpoints between them with the gas exchange thresholds.

**Figure 6:**
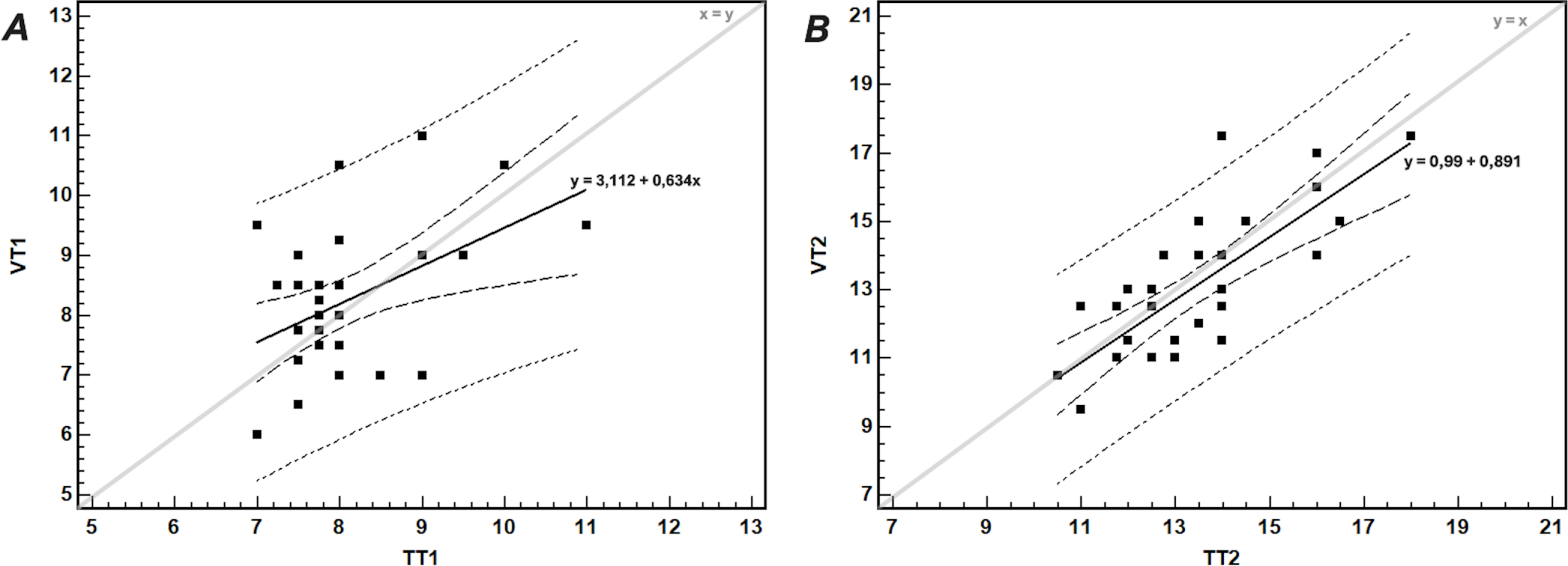
The relationship between (A) the first gas exchange (VT_1_) and temperature (TT_1_) thresholds, and (B) the second gas exchange (VT_2_) and temperature (TT_2_) thresholds. The thick continuous line shows the linear regression with corresponding equation; the short- and long-dashed lines show the 95% prediction and confidence intervals, respectively. The thin line shows the identity line.

The regression analysis for comparison of temperature with gas exchange thresholds is shown in Fig. 6.

The agreement between TT_2_ and VT_2_, tested by the Bland-Altman method, was satisfactory. After exclusion of an outlier (participant no. 8)*,* the mean difference between VT_2_ and TT_2_ was −0.47 ± 1.24 km/h, with 95% LoA −2.90–1.97 km/h. In 68% of participants the differences between TT_2_ and VT_2_ were within ± 1 SD of the mean difference (*i.e.,* within −1.71–0.77 km/h range). Only in four participants (12.5%) the differences were greater than 1.5 km/h.

### Test-retest reliability

Test-retest reliability for threshold values expressed as running speed was low (ICCs for VT_1_, TT_1_, VT_2_, and TT_2_ were 0.39, 0.18, 0.49, and 0.23, respectively). In some cases, the evaluators indicated a first-choice and an alternative (third) breakpoint/threshold value. In all such cases the indicated values matched among evaluators but were not always designated as the same level of choice. When these values were matched and included in analysis, the test-retest reliability was higher for VT_2_ (ICC = 0.93) but still showing poor reproducibility for TT_2_ (ICC = 0.36). The test-retest reliability was generally higher for threshold values expressed as VO_2_ (ICC = 0.52, 0.72, 0.69 and 0.68 for VT_1_, TT_1_, VT_2_ and TT_2_, respectively).

## Discussion

The aim of this study was to model the changes of core body (rectal) temperature during graded treadmill running to volitional exhaustion and to evaluate the relationship between CBT pattern with concurrent ventilatory and gas exchange pattern. The results confirmed our hypothesis that rectal temperature in young, fit men increases disproportionately with increasing running speed, showing threshold-like changes. Moreover, in most cases, two breakpoints in the CBT-speed relationship are present, and those thresholds are significantly correlated (TT_1_, moderately; TT_2_, strongly) to the first and second gas exchange threshold (VT_1_ and VT_2_, respectively).

The increase of rectal temperature in most of our participants followed a 3-phase model, while a two-segment model was detected in only three of our participants (Fig. 3), showing a single breakpoint that occurred at ∼70% of the maximal attained intensity (Figs. 3B, and 3C), similar to esophageal temperature thresholds for VE/VO_2_ and VE/VCO_2_ in the studies of White & Cabanac (1995) and White & Cabanac (1996).

Baseline temperature (37.20 ± 0.27 °C) and the highest temperature measured during the recovery of our participants (38.42 ± 0.38 °C) were also comparable, although higher, than the range of esophageal temperatures registered in the previous studies (White & Cabanac, 1995; White & Cabanac, 1996; Sancheti & White, 2006). Lower readings of esophageal *vs* rectal temperature during exercise in different environmental conditions were also described by previous studies (Mündel et al., 2016). A rectal temperature dip was observed in some of our participants at the beginning of the exercise. Small esophageal (blood) temperature dips (−0.2 °C) at the very beginning of a cycle ergometer test (White & Cabanac, 1995; Sancheti & White, 2006) were previously described and interpreted as a result of: (1) an increased limb perfusion causing increased return of cold blood from cutaneous circulation to the core, and (2) the heat produced and retained in active muscles for intramuscular warming, rather than being transported away from the muscles by blood (Alt et al., 1986; Alt, Stangl & Theres, 1993). Arguably, the stagnant or slow CBT rise in the moderate intensity range observed in some participants can be partly attributable to the lower temperature in the working muscles at the start of a graded running test, and the time needed for heat accumulation in the active muscles and equalization of muscle and core temperatures.

In the study comparing CBT with ventilatory parameters during graded cycling to exhaustion White & Cabanac (1995) described a disproportionate increase of both VE/VO_2_ and VE/VCO_2_ in relation to CBT. This onset of hyperventilation proportional to the increase in CBT at higher exercise intensities was described as a thermolytic response for selective brain cooling, from enhanced upper-airway evaporation and heat loss (White & Cabanac, 1995; White & Cabanac, 1996; Sancheti & White, 2006). Unfortunately, the authors did not report the CBT/workload relationship, hence giving no real evidence on the occurrence of a core temperature threshold with increasing exercise intensity. Moreover, the reported CBT threshold values were dependent upon the testing protocol (fast *vs* slow workload increase) and temperature probe placement (tympanic *vs* esophageal).

The premise that hyperventilation during high intensity activity contributes to selective brain cooling has also been contested, as decreasing arterial CO_2_ pressure (at intensities above AnT) contributes to cerebral hypoperfusion, thus diminishing heat removal and likely increasing brain temperature (Kety & Schmidt, 1948; Nybo et al., 2002; Nybo & Secher, 2011a; Nybo & Secher, 2011b). Therefore, it seems that metabolic factors, rather than brain cooling, primarily drive the disproportionate increase of both core temperature and ventilatory indexes during incremental exercise, following increased recruitment of fast twitch motor units at higher intensities (Nagata et al., 1981; Moritani et al., 1984; Airaksinen et al., 1992; Mateika & Duffin, 1994; Chwalbińska-Moneta, Hanninen & Penttila, 1994; Taylor & Bronks, 1994; Lucía et al., 1997; Lucía et al., 1999; Hug et al., 2003). Aaron et al. (1992) measured the O_2_ cost of hyperpnea during progressive exercise in healthy young subjects. From moderate to severe exercise, they noted an ∼80% increase in the oxygen cost per L of ventilation, while the average increase in total VO_2_ per step devoted to ventilation increased fivefold, from 8% to 39 ± 10%. The results of this study indicate that the decrease of ventilatory efficiency with increasing workload may considerably contribute to steeper CBT rise and the appearance of CBT thresholds. Meyer et al. (2004) demonstrated that metabolic acidosis induced by exercise is causally involved in the occurrence of hyperventilation at the respiratory compensation point (VT_2_). However, the VT_2_ in their study occurred even with complete buffering of metabolic acidosis, indicating that other physiological stimuli are included in the regulation of hyperpnea during intense exercise to volitional exhaustion.

Expressed as % VO_2max_, the average values of TT_1_ andTT_2_ in our participants were 56.8 ± 7.1% and 83.3 ± 10.0% VO_2max_, respectively. The average observed value was somewhat higher for TT_2_ than VT_2_ (Table 1), congruent with the results of Lucía et al. (1999) who reported higher values of the second EMG threshold for both *m. vastus lateralis* (86.9 ± 1.5% VO_2max_) and *m. rectus femoris* (88.0 ± 1.4% VO_2max_) compared with the values of VT_2_ (84.6 ± 6.5% VO_2max_). No statistically significant differences between the thresholds were found in that study (*p* > 0.05) (Lucía et al., 1999).

The objectivity of evaluators in determining TT_2_ was high, and threshold values were clearly identifiable in most participants. However, the existence of TT_1_ could not be firmly sustained due to poor agreement between the evaluators, as well as due to several cases (22%) in which this threshold could not be identified. In the study by Lucía et al. (1999) the first EMG threshold was identifiable in all 28 participants, although the authors did not state the level of agreement between evaluators in identification of the threshold. The low objectivity of the evaluation of TT_1_ indicates that the aerobic threshold cannot be reliably assessed from changes in CBT during a treadmill GXT. This might be influenced by the very low variability of VT_1_/TT_1_ and by discrete metabolic changes taking place at lower workloads, producing an unfavorable signal-to-noise ratio. In addition, the difficulty of TT_1_ assessment could result from the fact that the intensity at the aerobic threshold corresponds to the transition speed between walking and running gaits (Sentija & Markovic, 2009), a speed naturally avoided in locomotion (Minetti, Ardigo & Saibene, 1994).

The good agreement between TT_2_ and VT_2_ is comparable to the LoA (≈ 2–2.5 km/h) found between the second EMG and ventilatory thresholds (Lucía et al., 1999) as well as between the lactate and ventilatory thresholds (≈ 2–3 km/h) in the study of Gaskill et al. (2001) (approximate values, recalculated from original values reported in watts (Lucía et al., 1999) and ml O_2_/min (Gaskill et al., 2001)).

The mean difference between TT_2_ and VT_2_ (0.48 km/h, *p* = 0.029, Table 1) converted to time difference between the two equals 28.2 ± 74.4 s, with TT_2_ lagging behind VT_2_. This statistically significant difference between thresholds, defined as running speed, is negligible from a practical standpoint. TT_2_ and VT_2_ are two different indicators supposedly originating from the same underlying physiological processes and the time lag in their onset might be the consequence of their sequential occurrence and/or data latency. With increasing running speed, changes in some ventilatory variables like minute ventilation, tidal volume and breathing frequency are registered almost instantaneously. On the other hand, the change in CBT kinetics (increased rate of heat production within the active muscles) is registered with a delay for the blood transit time from the muscle capillaries of the locomotor and respiratory muscles to the temperature collection point (Dempsey, Harms & Ainsworth, 1996; Kalliokoski, Knuuti & Nuutila, 2004).

The high correlation between TT_2_ and VT_2_ is congruent with the results of previous studies that compared different methods for threshold detection, such as the study by Tikkanen et al. (2012), showing a high correlation between the EMG threshold and VT_2_ (*r* = 0.86, *p* < 0.001) and between the EMG threshold and the onset of blood lactate accumulation (*r* = 0.84, *p* < 0.001). Kang et al. (2014) compared anaerobic thresholds estimated by EMG and ventilatory parameters using different data filtering intervals (9, 15, 20, 25, 30 s) and detected a high correlation (*r* = 0.89−0.99) for different combinations of EMG and gas exchange data filtering.

The test-retest reliability found in the present study is lower than previously observed for EMG thresholds (ICC range 0.73–0.96 for the first and second EMG threshold) (Lucía et al., 1999) and for thresholds determined by the Dmax method (*r* = 0.78−0.93) (Cheng et al., 1992). On the other hand, our results are comparable to those of Dickhuth et al. (1999), who found a low reliability of epinephrine and norepinephrine thresholds (*r* = 0.49 and *r* = 0.46, respectively). The possible reasons behind the low test-retest reliability of the temperature thresholds determined in the present study include: small sample size; biological variability of the measured variables; error(s) caused by variation inherent to the measurement methods; error in subjective estimation (inter- and intra-evaluator), previously identified as potentially appreciable source of error (Gladden et al., 1985). Moreover, the results might have been influenced by the homogeneity of the sample of participants who repeated the test (showing above average fitness, and a narrow overall TT_2_ range), and by the suboptimal running speed resolution in the test (0.5 km/h).

### Limitations of the study

The findings of this study can only be generalized in reference to the characteristics of the sample, and therefore the conclusions drawn from the data are limited to healthy, active young men. Nevertheless, since the proposed causal physiological mechanism behind the occurrence of the thresholds (a steeper temperature increase at the transition from moderate to heavy, and from heavy to severe intensity) is metabolically driven and therefore supposed to be ubiquitous, we may presume the appearance of the temperature thresholds regardless of the participants’ characteristics. The study also failed to account for thermoregulatory factors like sweating and skin temperature regulation. To improve research findings, further studies should ensure representative samples with a broader range of participants, including both genders, various age groups and different fitness levels. The intra-evaluator reliability in detecting the temperature thresholds should also be tested.

Another methodological limitation of this study refers to the anatomical location for collection of CBT data. The use of tympanic and/or esophageal temperature was recommended in previous studies, as measurement sites more closely reflecting core (brain) temperature (White & Cabanac, 1995; White & Cabanac, 1996; Sancheti & White, 2006; Lim, Byrne & Lee, 2008), and, arguably, esophageal temperature also better reflected the increased passage of warm air through the adjacent airways. In line with our hypothesis, rectal temperature should more closely reflect the intensity-related increase in CBT, with a fast response due to the proximity of large pelvic and thigh muscles active in running, both *via* conductive (solid tissue) and convective (blood) heat transfer. The depth of insertion of the temperature probe was shallower than recommended by Hymczak et al. (2021), as it was chosen not to represent the best indication (highest value) of internal body temperature, but rather to be most reflective to rapid core temperature changes during incremental running. As shown in the study by Lee et al. (2010), T_re_ at shallower depths is most reflective of those rapid core temperature changes. Even so, and regardless of the strict procedure for insertion and securing of the rectal temperature probe, and the instructions given to the subjects regarding the depth of insertion (∼8 cm beyond the anal sphincter), we cannot exclude possible minor displacements of the temperature probe within the rectal area during measurement. Therefore, the location and variation of the depth of the rectal probe insertion may have contributed to a certain amount of variability of the measured temperature values (Lee et al., 2010) and the derived parameters.

## Conclusions

In conclusion, graded treadmill running induces a disproportionate increase in rectally measured core body temperature, with detectable breakpoints moderately (TT_1_) and highly (TT_2_) related to the first (aerobic) and second (anaerobic) ventilatory thresholds. Different patterns of rectal temperature increase were registered, with a 3-phase segmented linear regression model as the most common. Overall, smoother between-phase transitions and a higher signal-to-noise ratio was noted for CBT data, than for breath-by-breath gas exchange parameters. Highly objective assessment of the TT_2_, satisfactory agreement and correlation between TT_2_ and VT_2_ imply the presence of a temperature threshold that may be used in estimation of the anaerobic threshold. On the other hand, TT_1_ showed unsatisfactory reliability, lower objectivity and correlation with VT_1_. We presume that the same underlying physiological mechanisms account for the occurrence of gas exchange and core temperature thresholds with increasing workload. Namely, a decrease in locomotor and ventilatory efficiency, due to sequential motor unit recruitment pattern and hyperpnea, are manifested as thresholds delineating the moderate, heavy and severe intensity domains of physical activity. Further studies should be considered to elucidate the low reproducibility of the temperature thresholds described in this study, and to improve the methodology and enable practical implementation of core body temperature measurement for demarcation of exercise intensity domains. Future studies should also investigate the comparison of the temperature thresholds with other parameters used to assess the anaerobic threshold (EMG, blood lactate, *etc.*), and whether the findings in this study can be translated to diverse populations in regard to sex, age, fitness and acclimatization level, to modalities of graded exercise other than running (*i.e.,* cycling, walking, rowing, *etc.*), and test protocols of different total duration and workload increment.

##  Supplemental Information

10.7717/peerj.19686/supp-1 Supplemental Information 1 Ventilatory and temperature thresholds identified by three independent evaluators

## Abbreviations

AeT: aerobic (or lactate) threshold
AnT: anaerobic threshold
BF: breathing frequency
CBT: core body temperature
CO_2_: carbon dioxide
EMG: electromyography
GXT: graded exercise test
ICC: intraclass correlation coefficient
RCP: respiratory compensation point
T_re_: rectal (core body) temperature
TT_1_: first temperature threshold
TT_2_: second temperature threshold
TV: tidal volume
VE: minute ventilation
VCO_2_: carbon dioxide output
VO_2_: oxygen uptake
VT_1_: first gas exchange (ventilatory) threshold
VT_2_: second gas exchange (ventilatory) threshold

## Additional Information and Declarations

### Data Availability

The following information was supplied regarding data availability: The data are available in the Supplementary File ‘Data - evaluated thresholds’. The file shows the ventilatory and temperature threshold values identified by three independent evaluators. The values were used in the evaluation of the relationship between core body temperature pattern and concurrent ventilatory and gas exchange pattern during a graded treadmill test, as described in the manuscript.

## References

[ref-1] Aaron EA, Seow KC, Johnson BD, Dempsey JA (1992). Oxygen cost of exercise hyperpnea: implications for performance. Journal of Applied Physiology.

[ref-2] Airaksinen O, Remes A, Kolari PJ, Sihvonen T, Hānninen O, Penttilā I (1992). Real-time evaluation of anaerobic threshold with rms-EMG of working and nonworking muscles during incremental bicycle ergometer test. Acupuncture & Electro-Therapeutics Research.

[ref-3] Alt E, Hirgstetter C, Heinz M, Theres H (1986). Measurement of right ventricular blood temperature during exercise as a means of rate control in physiological pacemakers. Pacing and Clinical Electrophysiology.

[ref-4] Alt E, Stangl K, Theres H (1993). Central venous blood temperature. Rate adaptive cardiac pacing.

[ref-5] Altman DG, Bland JM (1983). Measurement in medicine: the analysis of method comparison studies. The Statistician.

[ref-6] Anderson GS, Rhodes EC (1989). A review of blood lactate and ventilatory methods of detecting transition thresholds. Sports Medicine.

[ref-7] Antonutto G, Di Prampero PE (1995). The concept of lactate threshold. A short review. The Journal of Sports Medicine and Physical Fitness.

[ref-8] Armstrong LE, Casa DJ, Millard-Stafford M, Moran DS, Pyne SW, Roberts WO (2007). Exertional heat illness during training and competition. Medicine & Science in Sports & Exercise.

[ref-9] Åstrand P-O, Rodahl K, Dahl HA, Strømme SB (2003). Textbook of work physiology: physiological bases of exercise.

[ref-10] Barnes KR, Kilding AE (2015). Running economy: measurement, norms, and determining factors. Sports Medicine—Open.

[ref-11] Binder RK, Wonisch M, Corra U, Cohen-Solal A, Vanhees L, Saner H, Schmid J-P (2008). Methodological approach to the first and second lactate threshold in incremental cardiopulmonary exercise testing. European Journal of Cardiovascular Prevention & Rehabilitation.

[ref-12] Bok D, Rakovac M, Foster C (2022). An examination and critique of subjective methods to determine exercise intensity: the talk test, feeling scale, and rating of perceived exertion. Sports Medicine.

[ref-13] Bosquet L, Léger L, Legros P (2002). Methods to determine aerobic endurance. Sports Medicine.

[ref-14] Brooks GA (1985). Anaerobic threshold: review of the concept and directions for future research. Medicine and Science in Sports and Exercise.

[ref-15] Buono MJ, Clancy TR, Cook JR (1984). Blood lactate and ammonium ion accumulation during graded exercise in humans. Journal of Applied Physiology.

[ref-16] Cheng B, Kuipers H, Snyder A, Keizer H, Jeukendrup A, Hesselink M (1992). A new approach for the determination of ventilatory and lactate thresholds. International Journal of Sports Medicine.

[ref-17] Chicharro JL, Legido JC, Alvarez J, Serratosa L, Bandres F, Gamella C (1994). Saliva electrolytes as a useful tool for anaerobic threshold determination. European Journal of Applied Physiology and Occupational Physiology.

[ref-18] Chwalbińska-Moneta J, Hanninen O, Penttila I (1994). Relationships between EMG and blood lactate accumulation during incremental exercise in endurance- and speed-trained athletes. Clinical Journal of Sport Medicine.

[ref-19] Chwalbinska-Moneta J, Krysztofiak H, Ziemba A, Nazar K, Kaciuba-Uściłko H (1996). Threshold increases in plasma growth hormone in relation to plasma catecholamine and blood lactate concentrations during progressive exercise in endurance-trained athletes. European Journal of Applied Physiology and Occupational Physiology.

[ref-20] Conconi F, Ferrari M, Ziglio PG, Droghetti P, Codeca L (1982). Determination of the anaerobic threshold by a noninvasive field test in runners. Journal of Applied Physiology.

[ref-21] Dempsey JA, Harms CA, Ainsworth DM (1996). Respiratory muscle perfusion and energetics during exercise. Medicine & Science in Sports & Exercise.

[ref-22] Dickhuth H-H, Yin L, Niess A, Röcker K, Mayer F, Heitkamp H-C, Horstmann T (1999). Ventilatory, lactate-derived and catecholamine thresholds during incremental treadmill running: relationship and reproducibility. International Journal of Sports Medicine.

[ref-23] Faude O, Kindermann W, Meyer T (2009). Lactate threshold concepts. Sports Medicine.

[ref-24] Frazão M, Silva PE, Cacau L de AP, Petrucci TR, Assis MC, Santos A da C, Brasileiro-Santos M do S (2021). EMG breakpoints for detecting anaerobic threshold and respiratory compensation point in recovered COVID-19 patients. Journal of Electromyography and Kinesiology.

[ref-25] Gaskill SE, Ruby BC, Walker AJ, Sanchez OA, Serfass RC, Leon AS (2001). Validity and reliability of combining three methods to determine ventilatory threshold. Medicine and Science in Sports and Exercise.

[ref-26] Gladden LB, Yates JW, Stremel RW, Stamford BA (1985). Gas exchange and lactate anaerobic thresholds: inter- and intraevaluator agreement. Journal of Applied Physiology.

[ref-27] González-Alonso J, Quistorff B, Krustrup P, Bangsbo J, Saltin B (2000). Heat production in human skeletal muscle at the onset of intense dynamic exercise. The Journal of Physiology.

[ref-28] Hug F, Faucher M, Kipson N, Jammes Y (2003). EMG signs of neuromuscular fatigue related to the ventilatory threshold during cycling exercise. Clinical Physiology and Functional Imaging.

[ref-29] Hymczak H, Gołąb A, Mendrala K, Plicner D, Darocha T, Podsiadło P, Hudziak D, Gocoł R, Kosiński S (2021). Core temperature measurement—principles of correct measurement, problems, and complications. International Journal of Environmental Research and Public Health.

[ref-30] Kalliokoski KK, Knuuti J, Nuutila P (2004). Blood transit time heterogeneity is associated to oxygen extraction in exercising human skeletal muscle. Microvascular Research.

[ref-31] Kang S-K, Kim J, Kwon M, Eom H (2014). Objectivity and validity of EMG method in estimating anaerobic threshold. International Journal of Sports Medicine.

[ref-32] Kaufmann S, Gronwald T, Herold F, Hoos O (2023). Heart rate variability-derived thresholds for exercise intensity prescription in endurance sports: a systematic review of interrelations and agreement with different ventilatory and blood lactate thresholds. Sports Medicine—Open.

[ref-33] Kety SS, Schmidt CF (1948). The effects of altered arterial tensions of carbon dioxide and oxygen on cerebral blood flow and cerebral oxygen consumption of normal young men. Journal of Clinical Investigation.

[ref-34] Kindermann W, Simon G, Keul J (1979). The significance of the aerobic-anaerobic transition for the determination of work load intensities during endurance training. European Journal of Applied Physiology and Occupational Physiology.

[ref-35] Lee J-Y, Wakabayashi H, Wijayanto T, Tochihara Y (2010). Differences in rectal temperatures measured at depths of 4–19 cm from the anal sphincter during exercise and rest. European Journal of Applied Physiology.

[ref-36] Lim CL, Byrne C, Lee JK (2008). Human thermoregulation and measurement of body temperature in exercise and clinical settings. Annals of the Academy of Medicine, Singapore.

[ref-37] Lind AR (1963). A physiological criterion for setting thermal environmental limits for everyday work. Journal of Applied Physiology.

[ref-38] Loat CER, Rhodes EC (1993). Relationship between the lactate and ventilatory thresholds during prolonged exercise. Sports Medicine.

[ref-39] Lucía A, Sánchez O, Carvajal A, Chicharro JL (1999). Analysis of the aerobic-anaerobic transition in elite cyclists during incremental exercise with the use of electromyography. British Journal of Sports Medicine.

[ref-40] Lucía A, Vaquero AF, Pérez M, Sánchez O, Chicharro JL, Sánchez V, Gómez MA (1997). Electromyographic response to exercise in cardiac transplant patients. Chest.

[ref-41] Mateika JH, Duffin J (1994). Coincidental changes in ventilation and electromyographic activity during consecutive incremental exercise tests. European Journal of Applied Physiology and Occupational Physiology.

[ref-42] Meyer T, Faude O, Scharhag J, Urhausen A, Kindermann W (2004). Is lactic acidosis a cause of exercise induced hyperventilation at the respiratory compensation point?. British Journal of Sports Medicine.

[ref-43] Meyer T, Lucía A, Earnest CP, Kindermann W (2005). A conceptual framework for performance diagnosis and training prescription from submaximal gas exchange parameters—theory and application. International Journal of Sports Medicine.

[ref-44] Minetti AE, Ardigo LP, Saibene F (1994). The transition between walking and running in humans: metabolic and mechanical aspects at different gradients. Acta Physiologica Scandinavica.

[ref-45] Moritani T, Tanaka H, Yoshida T, Ishii C, Yoshida T, Shindo M (1984). Relationship between myoelectric signals and blood lactate during incremental forearm exercise. American Journal of Physical Medicine.

[ref-46] Mündel T, Carter JM, Wilkinson DM, Jones DA (2016). A comparison of rectal, oesophageal and gastro-intestinal tract temperatures during moderate-intensity cycling in temperate and hot conditions. Clinical Physiology and Functional Imaging.

[ref-47] Myers J, Ashley E (1997). Dangerous curves. Chest.

[ref-48] Nagata A, Muro M, Moritani T, Yoshida T (1981). Anaerobic threshold determination by blood lactate and myoelectric signals. The Japanese Journal of Physiology.

[ref-49] Nybo L, Møller K, Volianitis S, Nielsen B, Secher NH (2002). Effects of hyperthermia on cerebral blood flow and metabolism during prolonged exercise in humans. Journal of Applied Physiology.

[ref-50] Nybo L, Secher NH (2011a). Counterpoint: humans do not demonstrate selective brain cooling during hyperthermia. Journal of Applied Physiology.

[ref-51] Nybo L, Secher NH (2011b). Rebuttal from Nybo and Secher. Journal of Applied Physiology.

[ref-52] Poole DC, Rossiter HB, Brooks GA, Gladden LB (2021). The anaerobic threshold: 50+ years of controversy. The Journal of Physiology.

[ref-53] Reggiani C, Bottinelli R, Stienen GJM (2000). Sarcomeric myosin isoforms: fine tuning of a molecular motor. Physiology.

[ref-54] Rusko H, Luhtanen P, Rahkila P, Viitasalo J, Rehunen S, Härkönen M (1986). Muscle metabolism, blood lactate and oxygen uptake in steady state exercise at aerobic and anaerobic thresholds. European Journal of Applied Physiology and Occupational Physiology.

[ref-55] Sancheti A, White MD (2006). Reproducibility of relationships between human ventilation, its components and oesophageal temperature during incremental exercise. European Journal of Applied Physiology.

[ref-56] Schneider D, McGuiggin M, Kamimori G (1992). A comparison of the blood lactate and plasma catecholamine thresholds in untrained male subjects. International Journal of Sports Medicine.

[ref-57] Schneider DA, Phillips SE, Stoffolano S (1993). The simplified V-slope method of detecting the gas exchange threshold. Medicine and Science in Sports and Exercise.

[ref-58] Sentija D, Markovic G (2009). The relationship between gait transition speed and the aerobic thresholds for walking and running. International Journal of Sports Medicine.

[ref-59] Skinner JS, Mclellan TH (1980). The transition from aerobic to anaerobic metabolism. Research Quarterly for Exercise and Sport.

[ref-60] Stienen GJ, Kiers JL, Bottinelli R, Reggiani C (1996). Myofibrillar ATPase activity in skinned human skeletal muscle fibres: fibre type and temperature dependence. The Journal of Physiology.

[ref-61] Sue DY, Wasserman K, Moricca RB, Casaburi R (1988). Metabolic acidosis during exercise in patients with chronic obstructive pulmonary disease. Chest.

[ref-62] Svedahl K, MacIntosh BR (2003). Anaerobic threshold: the concept and methods of measurement. Canadian Journal of Applied Physiology.

[ref-63] Taylor AD, Bronks R (1994). Electromyographic correlates of the transition from aerobic to anaerobic metabolism in treadmill running. European Journal of Applied Physiology and Occupational Physiology.

[ref-64] Tikkanen O, Hu M, Vilavuo T, Tolvanen P, Cheng S, Finni T (2012). Ventilatory threshold during incremental running can be estimated using EMG shorts. Physiological Measurement.

[ref-65] Wasserman K, Whipp BJ, Koyl SN, Beaver WL (1973). Anaerobic threshold and respiratory gas exchange during exercise. Journal of Applied Physiology.

[ref-66] Whipp BJ, Wasserman K (1970). Effect of body temperature on the ventilatory response to exercise. Respiration Physiology.

[ref-67] White MD, Cabanac M (1995). Core temperature thresholds for ventilation during exercise, temperature and ventilation. Advances in Experimental Medicine and Biology.

[ref-68] White MD, Cabanac M (1996). Exercise hyperpnea and hyperthermia in humans. Journal of Applied Physiology.

[ref-69] Yuan Y, So R, Wong S, Chan KM (2002). Ammonia threshold—comparison to lactate threshold, correlation to other physiological parameters and response to training. Scandinavian Journal of Medicine & Science in Sports.

